# 

**Published:** 1843-01

**Authors:** 


### BRITISH AND FOREIGN

### MEDICAL REVIEW

OR

### QUARTERLY JOURNAL

OF

### PRACTICAL MEDICINE AND SURGERY

LIBRARY OF THE
Orleans Parish Medical Society

EDITED BY

JOHN FORBES M.D. F.R.S. F.G.S.

---

VOL. XV.

JANUARY—APRIL 1843.

LONDON

JOHN CHURCHILL PRINCES STREET SOHO

MDCCCXLIII.

STATE OF LOUISIANA CHARITY HOSPITAL MEDICAL LIBRARY

C. ADLARD, PRINTER, BARTHOLOMEW CLOSE.

### BOOKS RECEIVED FOR REVIEW.

BRITISH.

1. The Evolution of Light from the Living Subject. By Sir Henry March, Bart. M.D. M.R.I.A. &c.—Dublin, 1842. 8vo, pp. 59.

2. An Easy Introduction to Chemistry. By George Sparkes. — London, 1841. 12mo, pp. 88. 3s. 6d.

3. A Series of Anatomical Sketches. By Wormald and M'Whinnie. Part V.

4. A Brief Account of Facts in reply to statements in the Memoir of Dr. Hope. By C. J. B. Williams, M.D. F.R.S. &c.—London, 1842. 8vo, pp. 16. 6*d*.

5. Lecture on the Elementary Composition of Food, considered in reference to their nutritive qualities. By J. Pereira, M.D. F.R.S.—London, 1842. 8vo, pp. 34.

6. Cerebral Physiology and Materialism, with the result of the application of animal magnetism to the cerebral organs. By W. C. Engledew, M.D.—London, 1842. 8vo, pp. 30. 1s.

7. Guy's Hospital Reports, No. XV. Oct. 1842. 6s.

8. Clinical Midwifery; with histories of 400 Cases of Difficult Labour. By R. Lee, M.D. F.R.S.—Lon. 1842. 12mo, pp. 224. 4s. 6d.

9. On a variety of False Aneurism. By Robert Liston, F.R.S. — London, 1842. 8vo, pp. 39.

10. Observations regarding Medical Education, in a letter addressed to the President of the College of Surgeons. By John Simon, Demonstrator of Anatomy in King's College, London.—Lon. 1842. 8vo, pp. 33.

11. Food, and its influence on Health and Disease. By M. Truman, M.D.—London, 1842. 8vo, pp. 240. 7s. 6d.

12. Retrospect of the progress of Medicine and Surgery for the year 1841-2. By Mr. E. O. Spooner and Mr. W. Smart. Read before the Southern branch of the Provincial Medical Association.—Blandford, 1842. 8vo, pp. 87. 3s.

13. A system of Practical Surgery. By William Fergusson, F.R.S.E. Professor of Surgery in King's College.—London, 1842. 8vo, pp. 596. 246 illustrations by Bagg. 12s. 6d.

14. An account of Askern and its mineral Springs. By Edwin Lankester, M.D. —London, 1842. 8vo, pp. 151. 4s. 6d.

15. On Injuries of the Head affecting the Brain. By G. J. Guthrie, F.R.S.—London, 1842. 4to, pp. 155. 6s.

16. Manual of Diseases of the Skin, from the French of MM. Cazenave and Schedel, with notes and additions. By Thomas Burgess, M.D. — London, 1842. 8vo, pp. 320. 7s.

17. Essays on Determination of Blood to the Head. By R. Hull, M.D.—London, 1842. 8vo, pp. 154. 5s.

18. A Treatise on the Principles and Practice of Homœopathy. By F. Black, M.D.—London, 1842. 8vo, pp. 239.

19. Turner's Chemistry. Seventh Edit. By Liebig and Gregory.—London, 1842. 8vo, pp. 1274. 28s.

20. Dr. Copland's Dictionary of Practical Medicine, Part viii.—Kidneys, Diseases of —Lungs, Diseases of.—1842. 4s. 6d.

21. Observations on the Admission of Medical Pupils to Bethlem Hospital. Third Edition, revised. By John Webster, M.D. —London, 1842. 8vo, pp. 62. 1s.

22. On the Preservation of the Health of Body and Mind. By Forbes Winslow, Surgeon.—Lond. 1842. 8vo, pp. 202. 7s. 6d.

23. The Natural History of Man. By J. C. Prichard, M.D. F.R.S. M.R.I.A. &c. With numerous Engravings on wood and steel.—London, 1843. 8vo, pp. 556. 30s.

24. Practical Observations in Midwifery, with Cases and Illustrations. By John Ramsbotham, M.D. Second Edit. revised. —London, 1842. 8vo, pp. 501. 12s.

25. Chemistry of Animal Bodies. By Thomas Thomson, M.D. F.R.S. &c.—Lond. 1843. 8vo, pp. 702. 16s.

26. The Retrospective Address, delivered at the Tenth Anniversary Meeting of the Provincial Medical and Surgical Association, held at Exeter, Aug. 3d and 4th, 1842. By James Black, M.D. &c. — Worcester, 1842. 8vo, pp. 146.

27. Account of a Case of Successful Amputation of the Thigh during the Mesmeric State. By W. Topham, Esq. and W. S. Ward, Esq. M.R.C.S.—London, 1842. 8vo, pp. 26. 1s.

28. Medical Reflections on the Water-cure. By J. Freeman, M.D. — London, 1842. 8vo, pp. 55. 1s. 6d.

29. The Great Physician, the Connexion of Diseases and Remedies with the Truths of Revelation. By John Gardner, Surgeon. —London, 1843. 8vo, pp. 359.

30. On the Chemical Discrimination of Vesical Calculi. By E. A. Scharling, Professor of Chemistry in the University of Copenhagen. Translated from the Latin, with an Appendix. By S. E. Hoskins, M.D. Plates.—Lond. 1842. 8vo, pp. 177. 7s. 6d.

31. A Practical Treatise on Pulmonary Consumption, its Pathology, Diagnosis, and Treatment, &c. By F. Cook, M.D. &c. —London, 1842. 8vo, pp. 120. 5s.

32. Recommendations for filling up the Register of Cases in Hospitals for the Insane.—Gloucester, 1842.

